# Railway By-Laws

**Published:** 1898-06-18

**Authors:** 


### The Hospital

A Journal of

The Medical Sciences and Hospital Administration.

Vol. XXIV.—No. 612.] Edited by Sir Henry Burdett, K.C.B., and Solomon C. Smith, M.D., M.R.C.P. [June 18, 1898.

### Railway By-laws.

At a time when everybody travels, and when journeys are frequently made by people in weak health, the issue of a new series of by-laws by one of the great railway companies, with an intimation that, when approved by the Board of Trade, they will be adopted by other companies also, is a matter which closely concerns the members of the medical profession, not only on their own account, but also on account of their patients. The by laws in question were published *in extenso* in the *Times* of Tuesday, with a commentary showing in what respects they differ from those at present in force, and the first impress ion produced by reading them is that they will make additional provision for the comfort and safety of travellers. It is said, moreover, as no opposition has been offered to them, that they will almost certainly be approved, an intimation which seems to imply that the Board itself will not feel bound to exercise any critical faculty which it may possess, and that the absence of expressions of disapproval from the public is of itself to be regarded as an indication of assent. If this be so, it is much to be wished either that the Board would take a different, and, as it appears to us, a more reasonable view of its duties, or else that the Railway Travellers' Protection Association, or some society or body representative of the travelling public, should make a careful examination of the proposals, in order to set forth in what respect they might be improved. We are much inclined to think that they are calculated to establish conditions in which "the reciprocity will be all on one side," and that they will leave the railway companies at full liberty to sanction breaches of them to any conceivable extent, although they may be enforced against offending passengers in a far more stringent manner than has hitherto been possible The saving clause, "except by express permission" of a guard or other servant of the company, occurs. more frequently than a due regard for the public convenience would allow. In no respect is this more conspicuous than with reference to a grievance of daily occurrence, the overcrowding of carriages. The new by-law provides that, when a carriage contains the full number of passengers which it is constructed to carry, no additional person shall enter or remain therein if requested by any passenger or by a guard or other authorised servant of the company not to do so. Any person infringing this by-law will be liable to specified penalties; and, on failure to quit the carriage on request by any guard or other servant or agent of the company may, without prejudice to the penalty, be removed therefrom by or under the direction of such guard, servant, or agent. We could mention many suburban lines on which the company habitually fails to provide sufficient carriage accommodation for the number of persons likely (as shown by daily recurring experience) to require places, with the result that third-class passengers force their way into first-class carriages, smokers into non-smoking carriages, and so on; and it is only too probable that this custom, which constitutes a very real and serious discomfort to many persons, will remain unchecked. The guard will say, "Oh, it is the last train," and will not require or enforce the exit of the intruders. The first part of the clause gives an appearance of authority to the aggrieved passenger, but this appearance is deceptive. Without the intervention of the guard, it does not extend to any power to clear the carriage of passengers in excess, although, if a passenger had complained, it is possible that the persons constituting the excess, if they could be identified and summoned, might be fined by a magistrate. If this be all, the remedy is at least as bad as the disease.

In the same sort of imperfect way there is an appearance of protection for female passengers. "Except by express permission of the guard of the train" no male person actually or apparently above eight years of age is to be allowed to travel in a carriage notified as being appropriated to the exclusive use of the female sex. We should fear that the "express permission" would always be given if a train were otherwise full, and that a small number of ladies will always be liable to be intruded upon by male passengers who would otherwise be in danger of being left behind. Offensive or indecent behaviour, and the use of bad language, will be more effectually provided against than they have been; and power will be given to the company's servants to exclude passengers whose persons or clothes are sufficiently dirty or offensive to constitute a nuisance, or to soil the linings of the carriages or the dresses of other passengers. We find no special mention of a common sin to which exception might be fairly taken, the sin, namely, of those who ride in first-class carriages and place their dirty boots on the cushions of the opposite seat, leaving a legacy of half-dried mud for any unfortunate person who may be compelled to come after them. On such people we should have no mercy, but we should greatly like to see travellers and the companies placed on a footing of more complete equality than at present seems likely to be attained, by depriving the latter of the sort of dispensing power which will enable them to render the most protective by-laws practically void and of no effect.

